# The value of health

**DOI:** 10.1186/1472-6963-8-136

**Published:** 2008-06-25

**Authors:** Wim Groot, Henriëtte Maassen van den Brink

**Affiliations:** 1 Department of Health Organization, Policy and Economics (HOPE), Maastricht University Faculty of Health, Medicine and Life Sciences, Maastricht, The Netherlands. Postoffice Box 616, 6200 MD Maastricht, The Netherlands; 2 "Scholar" Research Center for Education and Labor Market, University of Amsterdam Department of General Economics, Roetersstraat 11, 1018 WB Amsterdam, The Netherlands

## Abstract

**Background:**

A major problem in cost-effectiveness studies is where to draw the line between interventions which are cost-effective and those who are not. Lacking a notion about the value of a QALY, all ultimate values to the cost-effectiveness ratio are essentially arbitrary.

**Methods:**

This paper presents a simple empirical model to estimate the compensating income variation of diseases and health problems. The model is estimated using data for the Netherlands.

**Results:**

The compensating income variation is between €20,000 and €90,000. This is higher than most of the ultimate values used by policy-makers to decide whether an intervention is cost-effective. Our figures are roughly similar to those found in studies about the value of a statistical life year.

**Conclusion:**

Estimates on the compensating income variation of diseases and health problems may provide useful information on the maximum acceptable cost-effectiveness ratio of medical interventions than those currently used by policy makers.

## Background

Western countries spend an increasing share of income on health care. Where forty years ago western European countries and the United States spent 4–5% of GDP on health care, they nowadays spend 10% or more. The rapid increase in expenditures has fostered the need for more rational decision making in health care and hence the emergence of cost-effectiveness (CEA) and cost-utility analysis (CUA). A typical problem with both CEA and CUA, however, is where to draw the line between medical interventions which are welfare improving and those which are not. Some countries have informal rules about the maximum cost per QALY. In the Netherlands interventions with a cost per QALY of less than €20,000 are thought to be efficient. In Great Britain, the National Institute of Clinical Excellence (NICE) does not use a specific cost per QALY threshold above which a technology is rejected. However, NICE uses a band of approximately £20,000 – £30,000 "... as the threshold above which it would be increasingly likely to reject a technology on grounds of its cost-ineffectiveness" [[1]; p.2619]. Recently a Dutch government advisory board recommended putting the border at €80,000 per QALY [2]. All these figures are essentially arbitrary. What is required is a notion about how much individuals value a QALY or a life year saved. Only then can we decide whether medical interventions are truly welfare improving.

Few studies have tried to quantify the value of the health benefits obtained by medical technologies. These studies invariably conclude that the benefits of health care outweigh its costs. Murphy & Topel [3] find that the cumulative value of improvements in life expectancy in recent decades exceeds $3 trillion per year, much more than the annual health care expenditures. For an individual American, the increase in life expectancy in the twentieth century amounted to $1.2 million by the year 2000. Cutler & Richardson [4] estimate that – even if only a quarter of the overall improvement in health can be attributed to medical care – health care expenditures are worth the cost. Cutler & Meara [5] estimate that between 1950 and 1990 the costs of postnatal care increased by nearly $40,000 and the net benefits of treatment increased by nearly $200,000 per low-birth infant. Cutler & Huckman [6] estimate the costs and benefits of percutaneous transluminal coronary angioplasty (PTCA) and find that the net benefit is between $19,000 and $22,000 per additional PTCA.

Other studies have estimated the cost-benefit of medical expenditures by calculating the compensating income variation of health impairments, i.e. the income needed to make someone with a health impairment as well off as someone without in good health. Groot, Maassen van den Brink & Plug [7] and Groot & Maassen van den Brink [8,9] find that cardiovascular disease has a major negative impact on quality of life. These studies calculate that the compensating income variation is substantial and higher than the costs of most medical treatment for this disease. Groot & Maassen van den Brink [9] use data from the British Household Panel Survey and find that at the average values of household income, the compensating income variation of cardiovascular disease is approximately £49,000 for both men and women. These values are broadly similar to the amounts calculated for cardiovascular disease in Groot, Maassen van den Brink & Plug [7] using data for the Netherlands.

This paper builds on this research on separate diseases and uses data on subjective well-being to calculate the welfare effects – the compensating income variation – of various diseases and health impairments. Essentially the approach we take in this paper is based on an evaluation by respondents of their subjective well-being, individual level data on the prevalence of handicaps and diseases, and household income. Subjective well-being is assumed to depend – among others – on net household income and the health status. The estimation results are used to calculate the income-health trade-off, keeping well-being constant. This income-health trade-off – or compensating income variation – represents the monetary value of a health gain and can be interpreted as the willingness-to-pay for the elimination of a disease or handicap.

Measures of subjective well-being are widely used in psychology and social sciences, and increasingly also among economists. Krueger & Schkade [10] note that between 2000 and 2007 more than 150 papers and books have been published using data on life satisfaction or subjective well-being. The use of these direct measures of well-being is stimulated by findings that show that data on life satisfaction have good reliability [10] and validity [11,12]. However, questions about subjective well-being can be phrased in different ways. In some surveys respondents are asked how happy they are, others ask about satisfaction with life in general or with certain domains of life. Even if data on subjective well-being have good reliability and validity, the outcomes may be affected by the way these questions are phrased. In this paper we therefore use two different questions on subjective well-being: one about happiness and one about life-satisfaction. A comparison between the outcomes of these two will contribute to establishing the validity of our findings.

## Methods

The starting point of the empirical model is the subjective well-being function (W*). In the empirical application we operationalize subjective well-being by survey questions on happiness and life satisfaction. Well-being is assumed to be a cardinal variable. We feel justified in doing so by the findings of Ferrer-i-Carbonell & Frijters [13] who conclude that assuming ordinality or cardinality of happiness makes little difference. It is assumed that well-being of individual i is determined by income Y, health status H and other individual characteristics X:

(0.1) W_i_* = *W*(*Y_i_*, *H_i_*, *X_i_*)

where Y is the net annual household income. We assume that well-being is a linear function of the log of household income, health status and other individual characteristics:

(0.2) W_i_* = *β*_0 _+ *β*_1_*LogY_i_*+ *β*_2_*H_i_*+ *β*_3_*X_i_*+ *η_i_*

where β are coefficients that measure the impact of income and other characteristics on well-being and η is a normally distributed random error term capturing unmeasured and unmeasurable effects on well-being. The log of income is used instead of income itself in order to take account of the established fact of diminishing marginal utility of income (Using the log of income also provided more precise estimates of the income effect than using the level of income in our dataset) (see [14-18]).

The parameter estimates are used to calculate the compensating income variation (CIV) of health impairments, i.e. the additional amount of money needed to make someone with a health problem as well off as someone without these problems.

Someone with a health problem (H = 1) enjoys the same welfare as someone without these problems (H = 0) if:

(0.3) W*(*LogY*, *H_i_*= 0, *X_i_*) = W*(*LogY_i_*+ *LogCIV_i_*, *H_i_*= 1, *X_i_*)

Substitution in the specification of the equation for well-being above, this translates into:

(0.4) $$CIV_{i}=\exp(-\frac{\beta_{2}}{\beta_{1}})Y_{i}-Y_{i}$$

This gives the CIV as the increase in annual net household income.

The data are taken from the 2004 Periodic Life Style Survey (PLSS) of the Dutch Central Bureau of Statistics (The data are stored as file P1665 at the Data Archiving and Networked Services (DANS) [28]) The PLSS is an annual cross-sectional survey of the population of all ages in the Netherlands. The PLSS consists of various modules. All respondents (N = 21,706) receive a basic questionnaire containing demographic and socio-economic questions including income and questions about life situation (including happiness and life satisfaction). The questionnaires for the different modules of the survey are presented to a selection of the respondents. For our purposes we combine information from the basic questionnaire with the survey on health and labour (N = 11,117). The module on health and labour includes detailed information about the prevalence of diseases and health impairments. The response rate of the basic questionnaire is 59.9%, while the response rate of the health and labour module is 61.4%. We restrict the sample to people aged 16 and older and use information of respondents who have completed both the basic questionnaire and those on labour and health. This restricts the sample to approximately 6,800 respondents.

One advantage of using subjective well-being measures to calculate the CIV of health impairments is that the cognitive burden on respondents is lower than with other techniques. According to Diener & Suh [[12], p.437], 'When self-reports of well-being are correlated with other methods of measurement, they show adequate convergent validity.' Krueger & Schkade [10] draw similar conclusions for the reliability of subjective well-being measures. Diener & Suh [11] assert that the major advantage of the subjective well-being measures is '(...) that they capture experiences that are important to the individual' (p. 205). As a major disadvantage they note that 'although self-reported measures of well-being have adequate validity and reliability, it is naive to assume that every individual's responses are totally valid and accurate' (p. 206). Their review further shows that there is a high correlation between life satisfaction and a social index that includes cost of living, ecology, health, culture and entertainment, freedom and infrastructure indicators. Diener & Shuh [11,12] further assert that life satisfaction measures are found to be stable over time and across countries.

We use two measures of subjective well-being: one on happiness and one on life satisfaction. The happiness variable is defined by the response to the following survey question: "To what extent do you consider yourself a happy person?" there are five answering categories: Unhappy; Not so happy; Not happy, not unhappy; Happy; and Very happy. Life satisfaction is measured by the response to the following question: "To what extent are you satisfied with the life you now lead?" Again, there are five answering categories: Not so satisfied; Rather satisfied; Satisfied; Very satisfied; and Extremely satisfied.

A comparison between the happiness and life-satisfaction variable shows that the happiness variable has two negative answering categories (unhappy and not so very happy), while life satisfaction has only one (not so satisfied). The life satisfaction variable, on the other hand, has more positive options (very satisfied and extremely satisfied) than the happiness variable (very happy).

## Results

Table 1 contains the frequency distribution of the answers to the happiness and life satisfaction questions. Nearly 90% of all respondents say they are happy or very happy, while only less than 3% report to be not so very happy or unhappy. Similarly, more than 80% of all respondents say they are satisfied or very satisfied with their life and only less than 4% say there are not so satisfied. Differences between men and women in both happiness and life satisfaction ratings appear to be small.

**Table 1:** Frequency distribution happiness and life satisfaction variables

	*All*	*Men*	*Women*
*Happiness*			

Unhappy	0.4%	0.4%	0.4%
Not so very happy	2.5%	2.1%	2.8%
Not happy/not unhappy	7.6%	6.5%	8.6%
Happy	69.0%	71.3%	66.9%
Very Happy	20.6%	19.7%	21.3%
Mean value	4.056	4.069	4.043
# Observations	6792	3261	3531

*Life satisfaction*			

Not so satisfied	3.4%	3.3%	3.6%
Rather satisfied	8.0%	7.0%	8.9%
Satisfied	46.9%	48.8%	45.2%
Very satisfied	33.6%	33.1%	34.2%
Extremely satisfied	8.0%	7.9%	8.1%
Mean value	3.326	3.340	3.312
# Observations	6800	3264	3536

For the health impairment variables, H, the response to a battery of survey questions on health status is used. Table 2 presents the variable description and the sample means for the health variables, income and the control variables in the equations. The summary statistics in Table 2 show that 1.4% of the men and 1.3% of the women in our sample ever had cancer, while 2.1% of the men and 1.8% of the women have had heart problems during the past 12 months. The most prevalent health problems during the past 2 months are a cold or influenza and diarrhea. High blood pressure and serious back problems are the most prevalent health impairments during the past 12 months.

**Table 2:** Variable definition and sample means

	*Description*	*Men*	*Women*
Log income	Log annual household income	10.305	10.238
Age	Age	45.942	46.554
Marital status	Married or cohabiting	0.513	0.487
Urban area	Degree of urbanization	3.020	3.010
*Have you ever had:*			
Stroke	A celebral haemorrhage, celebral infarct or stroke	0.005	0.002
Heart attack	A heart attack	0.005	0.001
Cancer	Have you ever had cancer	0.014	0.013
Any diseases	Have had stroke heart attack or cancer	0.023	0.016
*Did you during the past 12 months have:*			
Heart problems	Heart problems	0.021	0.018
Migraine	Migraine or severe headache	0.082	0.193
Diabetes	Diabetes	0.042	0.035
High blood pressure	High blood pressure	0.108	0.141
Stricture of blood vessels	Stricture of blood vessels in belly or legs	0.023	0.020
Asthma, bronchitis	Asthma, chronic bronchitis, pulmonary emphysema, or CARA	0.071	0.073
Psoriasis	Psoriasis	0.024	0.017
Chronic eczema	Chronic eczema	0.036	0.042
Dizziness with falling	Dizziness with falling	0.020	0.034
Serious intestine problems	Severe or persistent intestine problems for more than 3 months	0.019	0.040
Involuntary incontinence	Involuntary incontinence	0.015	0.077
Serious back problems	Severe or persistent back problems	0.104	0.116
Wear out of the joints	Chronic wear out of joints	0.091	0.148
Arthritis	Arthritis	0.032	0.069
Problems with neck or shoulder	Condition of neck or shoulder	0.078	0.130
Problems elbow, wrist or hand	Condition of elbow, wrist or hand	0.052	0.083
Other long term disease	Other long term disease or condition	0.080	0.105
Any disease	Any of the diseases listed above during the past 12 months	0.469	0.589
*Did you during the past 2 months have:*			
Cold or influenza	A cold, influenza, etc.	0.382	0.412
Acute bronchitis	Acute bronchitis or pneumonia	0.018	0.021
Ear infection	Ear infection	0.027	0.026
Infection kidney or bladder	Infection of kidney or bladder	0.016	0.044
Diarrhea	Diarrhea	0.101	0.115
Vomiting	Vomiting	0.025	0.041
Ulcer	Ulcer	0.012	0.013
Any disease	Any of the diseases listed above during the past 2 months	0.462	0.504

Income refers to after tax household income. Net household income is computed by summing up all wage and non-wage income received by all income earners in the household.

The ordered probit estimates on happiness and life satisfaction are found in table 3. We present separate estimates for men and women and for the entire sample. The results show that only few of the health variables have a statistically significant effect on happiness or life satisfaction. Typically, we find no significant effects of major health problems as stroke, heart attack or cancer. We do, however, find that diabetes, dizziness with falling, influenza, problems with elbow, wrist or hand, and migraine have a substantially negative effect on both happiness and life satisfaction.

**Table 3:** Ordered probit estimation results on happiness and life satisfaction by gender

	*All*		*Men*		*Women*	
	*Happiness*	*Life satisfaction*	*Happiness*	*Life satisfaction*	*Happiness*	*Life satisfaction*

*Control variables*						
Male	-0.053 (0.030)	-0.077** (0.027)				
Log income	0.158** (0.026)	0.208** (0.024)	0.177** (0.039)	0.254** (0.035)	0.145** (0.035)	0.168** (0.033)
Age	-0.046** (0.005)	-0.036** (0.005)	-0.046** (0.007)	-0.044** (0.007)	-0.047** (0.007)	-0.031** (0.006)
Age^2^/100	0.039** (0.005)	0.034** (0.005)	0.040** (0.007)	0.042 (0.007)	0.038** (0.007)	0.029** (0.006)
Marital status	0.454** (0.036)	0.291** (0.033)	0.476** (0.055)	0.304** (0.050)	0.425** (0.048)	0.285** (0.044)
Urban area	-0.001 (0.011)	0.017 (0.010)	-0.005 (0.017)	0.006 (0.015)	0.003 (0.016)	0.027 (0.014)
*Health variables*						
Stroke	-0.428 (0.247)	0.092 (0.233)	-0.334 (0.295)	0.115 (0.276)	-0.533 (0.477)	0.092 (0.454)
Heart attack	0.284 (0.275)	-0.241 (0.252)	0.157 (0.305)	-0.351 (0.279)	0.909 (0.723)	-0.082 (0.645)
Heart problems	-0.155 (0.105)	-0.239** (0.097)	-0.278 (0.151)	-0.171 (0.139)	-0.000 (0.149)	-0.284* (0.138)
Cancer	-0.097 (0.121)	0.024 (0.112)	-0.013 (0.179)	0.145 (0.162)	-0.250 (0.168)	-0.118 (0.156)
Diabetes	-0.250** (0.075)	-0.203** (0.069)	-0.359** (0.104)	-0.204** (0.095)	-0.185 (0.110)	-0.212* (0.102)
Migraine	-0.166** (0.043)	-0.123** (0.039)	-0.120 (0.078)	-0.174* (0.071)	-0.180** (0.052)	-0.101* (0.048)
High blood pressure	-0.064 (0.046)	-0.057 (0.042)	-0.018 (0.070)	-0.037 (0.063)	-0.096 (0.060)	-0.071 (0.056)
Stricture of blood vessels	-0.128 (0.100)	-0.215** (0.093)	-0.278* (0.144)	-0.266* (0.132)	-0.006 (0.143)	-0.172 (0.133)
Asthma, bronchitis	0.081 (0.058)	0.016 (0.053)	0.057 (0.088)	0.006 (0.080)	0.115 (0.079)	0.048 (0.072)
Psoriasis	0.108 (0.098)	0.010 (0.090)	0.218 (0.137)	0.135 (0.123)	-0.027 (0.143)	-0.129 (0.133)
Chronic eczema	-0.035 (0.074)	-0.100 (0.068)	-0.018 (0.113)	-0.103 (0.102)	-0.092 (0.099)	-0.122 (0.092)
Dizziness with falling	-0.495** (0.091)	-0.297** (0.086)	-0.592** (0.158)	-0.417** (0.149)	-0.441** (0.113)	-0.225* (0.106)
Serious intestine problems	-0.174* (0.086)	-0.309** (0.080)	-0.170 (0.155)	-0.008 (0.142)	-0.188 (0.103)	-0.440** (0.096)
Involuntary incontinence	-0.100 (0.069)	-0.123* (0.063)	-0.134 (0.175)	-0.137 (0.161)	-0.062 (0.075)	-0.107 (0.070)
Serious back problems	-0.192** (0.047)	-0.244** (0.043)	-0.220** (0.071)	-0.253** (0.064)	-0.184** (0.064)	-0.243** (0.059)
Wear out of the joints	-0.029 (0.050)	-0.069 (0.045)	-0.015 (0.078)	-0.046 (0.071)	-0.044 (0.065)	-0.082 (0.060)
Arthritis	0.052 (0.069)	-0.004 (0.063)	-0.022 (0.123)	-0.113 (0.112)	0.105 (0.084)	0.058 (0.077)
Problems with neck or shoulder	-0.056 (0.051)	-0.064 (0.046)	0.009 (0.084)	0.016 (0.076)	-0.080 (0.064)	-0.108 (0.059)
Problems elbow, wrist or hand	-0.211** (0.060)	-0.214** (0.055)	-0.197* (0.097)	-0.216* (0.089)	-0.226** (0.076)	-0.212** (0.070)
Other long term disease	-0.139** (0.050)	-0.276** (0.046)	-0.158* (0.080)	-0.267** (0.073)	-0.119* (0.064)	-0.277** (0.059)
Cold or influenza	-0.097** (0.030)	-0.056 (0.028)	-0.095* (0.045)	-0.025 (0.040)	-0.105** (0.042)	-0.085* (0.039)
Acute bronchitis	-0.241* (0.107)	-0.180 (0.098)	-0.221 (0.167)	-0.347* (0.152)	-0.242 (0.140)	-0.051 (0.130)
Ear infection	0.097 (0.089)	0.121 (0.082)	0.339** (0.130)	0.226 (0.117)	-0.122 (0.124)	0.011 (0.115)
Infection kidney or bladder	0.025 (0.083)	-0.006 (0.076)	0.072 (0.168)	0.099 (0.153)	0.001 (0.096	-0.047 (0.088)
Diarrhea	-0.069 (0.048)	-0.050 (0.044)	-0.157* (0.072)	-0.070 (0.066)	-0.000 (0.066)	-0.027 (0.060)
Vomiting	0.017 (0.083)	-0.048 (0.076)	0.033 (0.147)	-0.179 (0.134)	0.039 (0.102)	0.019 (0.094)
Ulcer	-0.258* (0.125)	-0.130 (0.117)	-0.445* (0.184)	-0.316 (0.172)	-0.059 (0.173)	0.055 (0.165)
*Location points*						
α_1_	-2.420** (0.295)	-0.634 (0.262)	-2.189** (0.442)	-0.282 (0.387)	-2.575** (0.399)	-0.907** (0.359)
α_2_	-1.542** (0.287)	0.040 (0.261)	-1.296** (0.429)	0.349 (0.386)	-1.686** (0.390)	-0.192 (0.358)
α_3_	-0.834** (0.286)	1.541** (0.262)	-0.602 (0.428)	1.932** (0.387)	-0.960** (0.388)	1.245** (0.358)
α_4_	1.375** (0.287)	2.777** (0.263)	1.720** (0.429)	3.159** (0.389)	1.160** (0.388)	2.493** (0.360)
#observations	6792	6800	3261	3264	3531	3536
McFadden R^2^	0.049	0.035	0.048	0.035	0.052	0.039

* significant at 5% level; ** significant at 1% level.

This absence of a systematic effect of severe health impairments on subjective well-being is probably caused by the lack of sufficient 'positive' observations: there are too few respondents with these health problems to generate statistically significant results. To increase the precision of our estimates we have reduced the number of health conditions by running a factor analysis on the 27 health impairments variables. Nine factors were extracted. We have used these nine factors to define nine disease categories by including variables with a factor loading of 0.4 or more. The nine disease categories are defined as:

- Disease category 1 includes problems related to movement: serious back problems, wear out of the joints, arthritis, problems with neck or shoulder and problems with elbow, wrist or hand;

- Disease category 2 includes some chronic disease that increase the risk of cardiovascular disease: diabetes, high blood pressure and stricture of blood vessels;

- Disease category 3 are cardiovascular diseases: stroke, heart attack and heart problems;

- Disease category 4 includes: diarrhea and vomiting;

- Disease category 5 are respiration problems: asthma, bronchitis, cold or influenza and acute bronchitis;

- Disease category 6 includes cancer and intestine problems: cancer, serious intestine problems, other long term disease and infection of kidney or bladder;

- Disease category 7 includes: migraine, dizziness with falling and involuntary incontinence;

- Disease category 8 includes: ear infection and ulcer;

- Disease category 9 includes: psoriasis and chronic eczema.

Most of the factors combine diseases which are either related (f.e. psioriasis and chronic eczema) or can be symptoms of common causes (f.e. diarrhea and vomiting), or can have common consequences (f.e. diabetes and high blood pressure) or have in common that they provide serious discomfort or pain (f.e. ear infection and ulcer). The estimation results with dummy variables for these nine groups of health problems are in table 4.

**Table 4:** Ordered probit estimation results on happiness and life satisfaction by gender

	*All*		*Men*		*Women*	
	*Happiness*	*Life satisfaction*	*Happiness*	*Life satisfaction*	*Happiness*	*Life satisfaction*

*Control variables*						
Male	-0.066* (0.029)	-0.089** (0.027)				
Log income	0.162** (0.026)	0.212** (0.024)	0.181** (0.039)	0.257** (0.035)	0.149** (0.035)	0.174** (0.032)
Age	-0.046** (0.005)	-0.036** (0.005)	-0.046** (0.007)	-0.043** (0.007)	-0.046** (0.007)	-0.031** (0.006)
Age^2^/100	0.039** (0.005)	0.035** (0.005)	0.041** (0.007)	0.041** (0.007)	0.038** (0.007)	0.029** (0.006)
Marital status	0.457** (0.036)	0.289** (0.033)	0.485** (0.055)	0.301** (0.049)	0.421** (0.048)	0.280** (0.044)
Urban area	-0.000 (0.011)	0.019 (0.010)	-0.004 (0.017)	0.007 (0.015)	0.003 (0.015)	0.027* (0.014)
*Disease category variables*						
Disease 1	-0.197** (0.034)	-0.220** (0.031)	-0.180** (0.051)	-0.175** (0.046)	-0.204** (0.045)	-0.252** (0.042)
Disease 2	-0.142** (0.042)	-0.137** (0.038)	-0.177** (0.062)	-0.135** (0.056)	-0.115* (0.057)	-0.137** (0.053)
Disease 3	-0.153 (0.092)	-0.280** (0.085)	-0.240 (0.126)	-0.243* (0.115)	-0.049 (0.137)	-0.304* (0.127)
Disease 4	-0.109* (0.044)	-0.096* (0.040)	-0.191** (0.067)	-0.129* (0.061)	-0.049 (0.058)	-0.071 (0.054)
Disease 5	-0.099** (0.030)	-0.067* (0.027)	-0.084* (0.043)	-0.043 (0.039)	-0.114** (0.041)	-0.088* (0.038)
Disease 6	-0.158** (0.041)	-0.272** (0.038)	-0.169** (0.067)	-0.221** (0.061)	-0.154** (0.053)	-0.297** (0.049)
Disease 7	-0.216** (0.038)	-0.192** (0.035)	-0.240** (0.069)	-0.283** (0.063)	-0.199* (0.046)	-0.150** (0.042)
Disease 8	-0.100 (0.074)	-0.010 (0.069)	0.006 (0.108)	-0.006 (0.098)	-0.189 (0.103)	-0.012 (0.096)
Disease 9	0.031 (0.061)	-0.059 (0.056)	0.128 (0.090)	0.032 (0.081)	-0.064 (0.083)	-0.143 (0.077)
*Location points*						
α_1_	-2.345** (0.293)	-0.601* (0.261)	-2.084** (0.438)	-0.217 (0.385)	-2.497** (0.395)	-0.869* (0.356)
α_2_	-1.500** (0.286)	0.063 (0.261)	-1.252** (0.427)	0.405 (0.385)	-1.638** (0.387)	-0.167 (0.355)
α_3_	-0.802** (0.285)	1.552** (0.261)	-0.572 (0.426)	1.975** (0.386)	-0.922* (0.385)	1.255** (0.356)
α_4_	1.392** (0.285)	2.783** (0.262)	1.732** (0.426)	3.193** (0.387)	1.180** (0.386)	2.499** (0.357)
						
#observations	6866	6874	3291	3294	3575	3580
McFadden R^2^	0.044	0.032	0.041	0.030	0.048	0.035

* significant at 5% level; ** significant at 1% level. Disease category 1: serious back problems, wear out of the joints, arthritis, problems with neck or shoulder and problems with elbow, wrist or hand; Disease category 2: diabetes, high blood pressure and stricture of blood vessels; Disease category 3: stroke, heart attack and heart problems; Disease category 4: diarrhea and vomiting; Disease category 5: asthma, bronchitis, cold or influenza and acute bronchitis; Disease category 6: cancer, serious intestine problems, other long term disease and infection of kidney or bladder; Disease category 7: migraine, dizziness with falling and involuntary incontinence; Disease category 8: ear infection and ulcer; Disease category 9: psoriasis and chronic eczema.

We now find that almost all disease categories have a statistically significant and negative effect on well-being. The only exceptions are ear infection and ulcer (category 8) and psoriasis and chronic eczema (category 9). Cancer and intestine problems (category 6) and migraine and dizziness (category 7) have the largest negative effects on well-being.

The log of household income has a statistically significant and positive effect on subjective well-being, but the income effects are relatively small. We find that the income elasticity with respect to life satisfaction is higher than the income elasticity of happiness. In the average values of both variables, the total sample income elasticity of happiness is 0.043 and the income elasticity of life satisfaction is 0.120, indicating that a 10% increase in net household income increases happiness by 0.43% and life satisfaction by 1.2%. We further find that income elasticities are larger for men than for women, although the differences in income effects between men and women are all within one standard error from eachother. Income elasticities for happiness are 0.048 for men and 0.040 for women, while income elasticities of life satisfaction are 0.139 for men and 0.104 for women.

We further find a statistically significant and negative effect of being male on life satisfaction but not on happiness. Age has a U-shaped effect on well-being. Both for the happiness variable and the life satisfaction variable the bottom of the age parabola is at age 60. Finally, being married or living together with a partner has a positive and substantial effect on subjective well-being.

We use the estimation results of table 4 to calculate the CIV for every individual in our sample. Table 5 contains the calculated sample means of the CIV of health impairments.

**Table 5:** Compensating income variation for health impairments (in € per year)

	*All*		*Men*		*Women*	
	*Happiness*	*Life satisfaction*	*Happiness*	*Life satisfaction*	*Happiness*	*Life satisfaction*

Disease 1	79,481	61,032	57,031	32,670	98,168	109,011
Disease 2	46,962	30,413	55,543	23,135	38,963	40,097
Disease 3	52,614	91,951	92,606	52,707	13,037	158,643
Disease 4	32,136	19,177	62,702	21,828	13,037	16,871
Disease 5	28,208	12,445	19,773	6,098	38,478	22,039
Disease 6	55,312	87,306	51,694	45,636	60,638	151,066
Disease 7	93,539	49,339	92,606	67,222	93,823	45,806
Disease 8	28,590	1,617	-1,092	791	85,560	2,390
Disease 9	-5,831	10,744	34,429	-3,920	17,965	42,679

Disease category 1: serious back problems, wear out of the joints, arthritis, problems with neck or shoulder and problems with elbow, wrist or hand; Disease category 2: diabetes, high blood pressure and stricture of blood vessels; Disease category 3: stroke, heart attack and heart problems; Disease category 4: diarrhea and vomiting; Disease category 5: asthma, bronchitis, cold or influenza and acute bronchitis; Disease category 6: cancer, serious intestine problems, other long term disease and infection of kidney or bladder; Disease category 7: migraine, dizziness with falling and involuntary incontinence; Disease category 8: ear infection and ulcer; Disease category 9: psoriasis and chronic eczema.

In general we find that the CIV of health problems based on the happiness estimates are larger than those calculated from the life satisfaction estimates. The CIV for women are also mostly larger than those for men. The highest CIV are for movement problems (category 1), cardiovascular problems (category 3), cancer and intestine problems (category 6) and for migraine and dizziness (category 7).

## Discussion

We find that results for both measures of subjective well-being – happiness and life satisfaction – are roughly similar. Although for a few disease categories estimates vary widely (f.e. for cardiovascular disease among women the CIV based on the happiness estimates is much smaller than the corresponding life satisfaction estimate). The correspondence between the happiness and life satisfaction estimates gives some confidence in the reliability of the results. Most effects – for example the income effects – are more pronounced on life satisfaction than on happiness however.

Income has a statistically significant and positive effect on life satisfaction for both men and women, as expected. The size of the effect of income on life satisfaction is rather small, however. This also explains the relatively large values for the compensating income variation for health impairments (see the formulae for the calculation of the CIV: a smaller value for β_1 _increases the size of the CIV).

Because of small cell sizes, we were unable to calculate the compensating income variation for individual diseases and health problems. Larger data-sets are needed to draw conclusions on separate diseases and health impairments. We therefore used factor analysis to classify diseases in nine broader categories. Unfortunate this may seem, however for our recommendations about the ultimate values of the cost-effectiveness of medical intervention this is of minor importance as policy makers are probably more interested in values for broad categories of diseases rather than have separate values for every single disease and health problem.

We find that the CIV is approximately €20,000–90,000 depending on the disease category and the measure of subjective well-being used. These figures suggest that cost-effective medical interventions are interventions with a cost per QALY of less than €20,000–90,000. These figures are higher than the ultimate values used by policy makers in the Netherlands – where the upper-bound of acceptable cost-effectiveness ratio varies between €18,000 and €20,000 per QALY [19] – and the United Kingdom – where the upper-bound is approximately £30,000 or £43,000 [20] – to decide whether interventions are still cost-effective.

It should further be noted that few medical interventions are able to completely cure a disease. If we account for efficacy and the probability that a treatment provides perfect cure, the discrepancy between the CIV and the maximum acceptable costs per QALY used by policy makers in their decisions whether interventions are cost-effective becomes even larger.

How valid are these estimates? A much used measure for the value of a QALY is Laupacis et al [21]. This study argues that the value of a QALY is about $100,000. In a meta-analysis of 33 studies that have calculated the value of a statistical life, Mrozek & Taylor [22] infer that the value of statistical life is $1.5–2.5 million. At a 5% discount rate this would make the value of a statistical life year between $76,500 and $127,500. Viscusi [23,24] summarizes 24 studies and concludes that the appropriate range for the value of a statistical life is $4–9 million, as this is the range in which most estimates lie. In a regression of 'best estimates' of values of a statistical life year on a number of characteristics of the studies and countries included, Miller [25] finds that the value of a statistical life year is $4 million. Moore & Viscusi [26] estimate that the value of a statistical life is $5 million. From this latter finding it can be calculated that the properly discounted value of a statistical life year is about $230.000.

Our estimates are roughly similar to the value of a QALY inferred by Laupacis et al [21] and the value of a statistical life year estimates by Mrozek & Taylor [22], but lower than those calculated from Miller [25], Moore & Viscusi [26] and Viscusi [23,24].

It should be noted, however, that the theoretical assumptions on which the figures on the maximum acceptable costs per QALY as used by policy makers, the value of a QALY, the value of a statistical life year and the estimates of the compensating income variation are based differ. This limits the comparability of the different values. The value of a statistical life year and the compensating income variation are based on strictly welfarist assumptions, whereas extra welfarist arguments may influence the value of a QALY and the maximum acceptable costs per QALY used by health policy makers. Furthermore, the value of a statistical life year is usually derived from observed risk-taking behavior – f.e. through the compensating wage differential for work-related health hazards or the willingness to pay for safety measures – and are usually not conditioned on health status.

In our analysis we have ignored the possibility of adaptation and scale of reference bias in the evaluation of subjective well-being. Adaptation refers to the notion that the effect of a disease on subjective well-being diminishes with the duration of the disease, while scale of reference bias refers to the notion that people compare themselves with others – for example people of the same age or people with the same health problems – in evaluating their well-being (see [27]). Also, the possibility that some respondents may be more optimistic while others may be more pessimistic in the face of a disease or health problem has been ignored. For example, Groot & Maassen van den Brink [9] find that that men are relatively more optimistic and less pessimistic than women in their evaluation of subjective well-being questions. This study further finds that cardiovascular disease makes people both less optimistic and less pessimistic.

## Conclusion

Cost-effectiveness studies are increasingly used to decide whether new medical technologies are valuable enough to merit reimbursement and inclusion in a standard package of medical services. However, cost-effectiveness studies are only valuable if we know where to draw the line between interventions which are cost-effective and those who are not. The maximum acceptable cost per QALY used to decide whether a medical intervention is worthwhile is primarily a political decision. If, for political reasons, total public expenditures on health care are limited, a rational allocation of these limited resources may require a relatively low maximum acceptable cost per QALY level. However, from a welfare point of view insight in the value individuals attach to health and health improvements may be useful to determine the maximum acceptable cost per QALY for a medical intervention (and, by implication, the total resources available for health care). This paper has estimated the monetary value of health by the compensating income variation for health impairments. The results suggest that the ultimate values of cost-effectiveness ratios used in some countries are lower than the value people attach to their health.

## Competing interests

The authors declare that they have no competing interests.

## Authors' contributions

WG participated in the design of the study, performed the statistical analysis and drafted the manuscript. HMvdB participated in the design of the study. All authors read and approved the final manuscript.

## Pre-publication history

The pre-publication history for this paper can be accessed here:


